# Correction: Mierzejewska, Z.A. Effect of Laser Energy Density, Internal Porosity and Heat Treatment on Mechanical Behavior of Biomedical Ti6Al4V Alloy Obtained with DMLS Technology. *Materials* 2019, *12*, 2331

**DOI:** 10.3390/ma12182928

**Published:** 2019-09-10

**Authors:** Żaneta Anna Mierzejewska

**Affiliations:** Faculty of Mechanical Engineering, Bialystok University of Technology, Wiejska 45c Street, 15-351 Białystok, Poland; a.mierzejewska@pb.edu.pl

The authors wish to make the following correction to this paper [1]: The author name “Mierzejewska Żaneta Anna” should be “Żaneta Anna Mierzejewska”.

We apologize for any inconvenience caused to the readers.
